# Conformations of the Pyranoid Sugars. IV. Infrared Absorption Spectra of Some Fully Acetylated Pyranoses

**DOI:** 10.6028/jres.065A.030

**Published:** 1961-06-01

**Authors:** R. Stuart Tipson, Horace S. Isbell

## Abstract

The infrared absorption spectra of twenty pyranose acetates in the range of 5000 to 250 cm^−1^ are reported. The conformation adopted by each of fourteen of the corresponding methyl glycopyranosides (or their acetates) had previously been assigned by us from a study of their infrared spectra. Analysis of the spectra revealed, for the pyranose acetates (as for the methyl glycopyranosides and their acetates), groups of absorption bands which showed a concerted shift on change of anomeric disposition. Assignment of conformation by the methods earlier developed indicated that, for most of the compounds examined, the conformation is the same for the pyranose acetate, methyl glycopyranoside, and acetylated methyl glycopyranoside of one anomer of a monosaccharide.

The following new assignments of conformation were made: the CA conformation for penta-*O*-acetyl-*α*-l-*xylo*-hexulopyranose and hexa-*O*-acetyl-*α*-d-*gluco*-heptulopyranose; and, possibly, either a non-chair conformation or a mixture of the CA and CE conformations for hexa-*O*-acetyl-l-*glycero*-*β*-d-*gluco*-heptopyranose and hexa-*O*-acetyl-d-*glycero*-*β*-d-*galacto*-heptopyranose.

## 1. Scope and Purpose of the Project

As the first objective of the present project, the infrared absorption spectra of a variety of fully acetylated pyranoses were recorded for use in identifying supposedly identical samples. The spectra of three of the compounds included in the present study were recorded by Kuhn [1]^1^ for a limited range (1250 to 667 cm^−1^). In the following year, the spectra of 17 of the compounds were given (for the range of 5000 to 667 cm^−1^) by Isbell and coworkers in a privately circulated report [2] which was subsequently published [3]. Fifteen of the spectra obtained by these investigators were later discussed by Whistler and House [4], who noted certain bands in the range of 1170 to 931 cm^−1^. Next, Barker and coworkers [5] examined the spectra of two of Kuhn’s compounds and of the enantiomorph of one, together with those of three others that are included in the present study. These workers [5] discussed some of the bands in the range of 960 to 730 cm^−1^, but the spectra were published in insufficient detail to permit comparison over a wide spectral range. In the present article, the infrared absorption spectra of 20 fully-acetylated pyranoses, in the range of 5000 to 250 cm^−1^, are given; all of the bands of all of these esters have been measured and have received consideration.

The second objective was the discovery of correlations that might be of value in structural analysis, both as regards (a) the presence of certain functional groups and (b) the particular conformation assumed by each ester. Isbell and coworkers [3] recorded the infrared spectra of 17 of the esters dissolved in suitable solvents, and were able to reach certain conclusions regarding correlations of the kind mentioned. However, at the low concentrations they employed, minor bands were absent or difficult to detect. The spectrograms have now been recorded for the solid phase, by use of pellets consisting of crystalline ester suspended in an alkali-metal halide.

In parts II and III of this series [6, 7], a method was described for gaining information regarding the conformations of aldopyranosides and their acetates from analysis of their infrared spectra. The analysis revealed groups of absorption bands, characteristic for each group-configuration, which displayed a concerted shift on change of anomeric disposition. This empirical observation was employed, in conjunction with a consideration of instability factors (arrived at on theoretical grounds), in making conformational assignments and in deciding the arrangement of groups (e.g., axial or equatorial) at the anomeric carbon atom of these compounds. The present article describes the results obtained on applying the same kind of analysis to the infrared spectra of (a) 14 fully-acetylated aldopyranoses related to 13 of the methyl aldopyranosides and to 12 of the acetylated methyl aldopyranosides previously studied, and (b) four esters whose corresponding methyl glycopyranoside or its acetate had not been included in parts II and III. Because of a lack of configurationally related esters, the conformations of two of the acetylated pyranoses could not be determined.

## 2. Compounds Investigated

Table 1 gives a list of the compounds, their code numbers [8], and an index to the spectrograms; the serial number of a compound is the same as the number of its spectrogram. Also included in table 1 are (a) the conformation (where known) of the corresponding methyl glycoside and acetylated methyl glycoside, as determined in parts II and III of this series [6, 7], and (b) assignments of conformations to the acetylated pyranoses. The conformations are indicated by the system devised by Isbell and Tipson [9].

The spectra were measured in the region of 5000 to 250 cm^−1^. The spectrograms are given together with a discussion of (a) the structure of the compounds and (b) some of the outstanding features of their spectra.

All of the compounds listed in table 1 are fully acetylated monosaccharides, and all have the pyranoid ring. Compounds 5 and 8 are acetates of ketopyranoses; the rest of the compounds are acetates of aldopyranoses. The pyranose acetates differ in regard to one or more of the following structural features: (a) the *α* or *β* anomeric configuration at the reducing carbon atom; (b) the configurations of the other carbon atoms of the pyranoid ring (including C5 in the aldohexose and the heptose acetates); (c) the nature of the substituent, if any, at C5 (including the configuration at C6 of the aldoheptose acetates); and (d) the presence or absence of a *C*-(acetoxymethyl) group at C1 of the pyranoid ring.

## 3. Reference Aldopyranosides and Their Acetates (of Known Conformation)

In part II of this series, the stable conformations of 13 crystalline aldopyranosides and, in part III, of 12 crystalline acetates thereof, all of which are related to compounds in the present study, were deduced from analysis of their infrared spectra.

In the present study, the crystalline acetates of 14 corresponding pyranoses were available (group A). In addition, six pyranose acetates (group B) whose glycosides or acetylated glycosides had not been available were examined. The spectra of the acetates in group A were analyzed, in order to determine whether they were explicable on the basis that a pyranose acetate has the same conformation as its glycopyranoside and acetylated glycopyranoside relatives. The conclusions drawn from this study were then applied to deducing the stable conformation of members of group B.

## 4. Classification of the Acetylated Pyranoses into Configurationally Related Groups

The 20 esters were classified into four groups; the members of each group have like configurational features.

### 4.1. Acetylated Pyranoses of the *xylo* Configuration

The members of this group have general formula I for the CA conformation.

**Figure:**
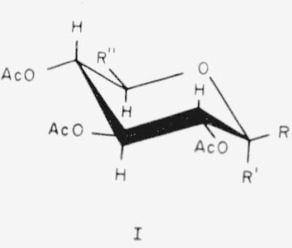


The following compounds, in this conformation, have structure I, with the substituents indicated.

1. Tetra-*O*-acetyl-*α*-d-xylopyranose, R=H; R′ = OAc; and R″=H.
2. Tetra-*O*-acetyl-*β*-d-xylopyranose, R = OAc: R′ = H; and R″=H.
5. Penta-*O*-acetyl-α-l-*xylo*-hcxulopyranose (penta-*O*-acetyl-*α*-l-sorbopyranose), R = CH_2_OAc; R′ = OAc; R″=H; and the molecule is the mirror image of that depicted.
6. Penta-*O*-acetyl-*α*-d-glucopyranose, R = H; R′ = OAc; and R″ = CH_2_OAc.
7. Penta-*O*-acetyl-*β*-d-glucopyranose, R = OAc; R′ = H; and R″ = CH_2_OAc.
8. Hexa-*O*-acetyl-*α*-d-*gluco*-heptulopvranose, R = CH_2_OAc; R′ = OAc; and R″ = CH_2_OAc.
9. Hexa - *O* - acetyl - l - *glycero* - *β*-d-*gluco* - heptopyranose, R = OAc; R′ = H; and R = 
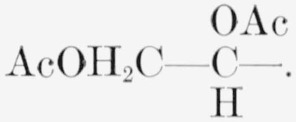
.

Compound 10 has the following formulas (II) for the two chair-conformations.

**Figure:**
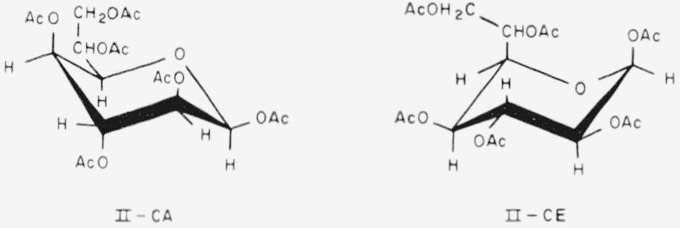

10. Hexa-*O*-acetyl-d-*glycero*-*β*-d-*ido*-heptopyranose

### 4.2. Acetylated Pyranoses of the *lyxo* Configuration

Four members of this group have the *lyxo* or *manno* configuration and general formula III for the CA conformation.

**Figure:**
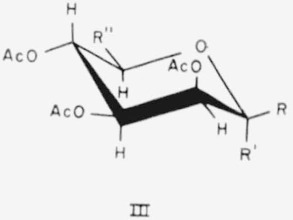


The following compounds, in this conformation, have structure III, with the substituents indicated.

3. Tetra-*O*-acetyl-*α*-d-lyxopyranose, R=H; R′ = OAc; and R″=H.
11. Penta-*O*-acetyl-*α*-d-mannopyranose, R = H; R′ = OAc; and R″ = CH_2_OAc.
12. Penta - *O* - acetyl - *β* - d - mannopyranose, R = OAc; R′ = H; and R″ = CH_2_OAc.
13. Hexa - *O* - acetyl - d- *glycero* - *α* - l - *manno* - heptopyranose, R=H; R′ = OAc; R″= H 
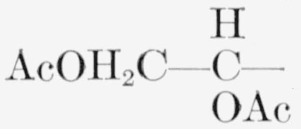
; and the molecule is the mirror image of that depicted.

Compounds 14 to 16 have the d-*gulo* configuration and the following general formula (IV) for the CA conformation.

**Figure:**
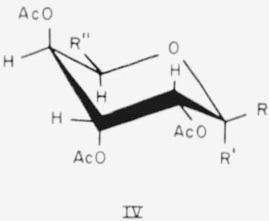

14. Penta-*O*-acetyl-*α*-d-gulopyranose, R = H; R′= OAc; and R″=CH_2_OAc.
15. Hexa - *O* - acetyl - d - *glycero* - *α* - d - *gulo* - heptopyranose, R = H; R′ = OAc; and R″= 
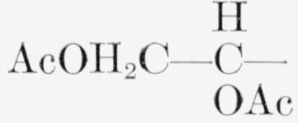
.
16. Hexa - *O* - acetyl - d - *glycero* - *β* - **d** - *gulo* - heptopyranose, R = OAc; R′ = H; and R″= 
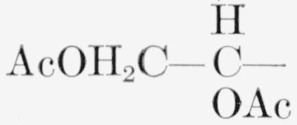
.

### 4.3. Acetylated Pyranoses of the *arabino* Configuration

The CE conformation of compound 4 and the CA conformation of compounds 17 to 19 are depicted in general formula V.

**Figure:**
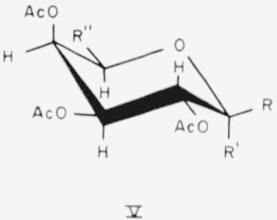

4. Tetra-*O*-acetyl-α-l-arabinopyranose, R = OAc; R′=H; and R″=H.
17. Penta - *O* - acetyl - *α* - d - galactopyranose, R = H; R′-OAc; and R″ = CH_2_OAc.
18. Penta - *O* - acetyl - *β* - d - galactopyranose, R = OAc; R′=H; and R″ = CH_2_OAc.
19. Hexa - *O* - acetyl - d - *glycero* - *β* - d - *galacto* - heptopyranose, R = OAc; R′=H; and R″ = 
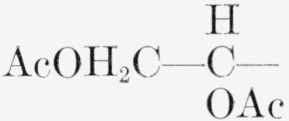
.

### 4.4. Acetylated Aldopyranose of the *ribo* Configuration

The two chair-conformations of compound 20 are depicted in formulas VI.

**Figure:**
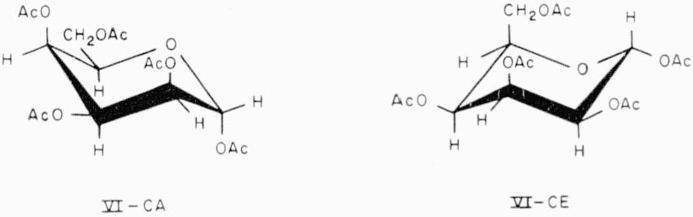

20. Penta-*O*-acetyl-*α*-d-talopyranose

## 5. Discussion of the Spectra

In the present study, the positions of the various absorption bands for each of 20 acetylated pyranoses have been determined; the relative intensities of absorption were not examined in detail. The bands were compiled, and were studied by comparative methods, as previously described [10].

The conformations of 13 of the corresponding glycopyranosides and of 12 of the acetylated glycopyranosides had previously been assigned by us [6,7] from a study of their infrared absorption spectra (see table 1). Assignment was made on the basis of the empirical observation that a change of anomeric disposition was accompanied by a shift of certain absorption bands.

The same kind of examination has now been applied to the spectra of the fully acetylated derivatives of 14 pyranoses related to the glycosides just mentioned, and a similar empirical relationship (between anomeric disposition and the position of certain absorption bands) was observed. On the basis of this finding, conformational assignments were made for these 14 pyranose acetates and were found to be in essential agreement with those for their glycosidic relatives (both acetylated and unacetylated).

The remaining 6 spectra were those of pyranose acetates for whose glycosidic relatives we had made no conformational assignment. The spectra of four of these acetates (compounds 5, 8, 9, and 19 in table 1) were intercompared with those of related acetates, especially in regard to anomer-differentiating absorption bands, and assignments were made (see table 1). For compounds 10 and 20, the spectra of related acetates were not available, and assignments could not be made.

### 5.1. Absorption Bands Possibly Indicative of the Disposition of Groups at the Anomeric Carbon Atom of the Tetra-*O*-acetyl-pentopyranoses

The spectrum of tetra-*O*-acetyl-*β*-d-xylopyranose (compound 2) was compared with that of its *α* anomer (compound 1), in order to determine the effect of changing the anomeric acetoxyl group from equatorial to axial (or vice versa). A comparison was then made with the spectrum of tetra-*O*-acetyl-*α*-l-arabinopyranose (compound 4).

In table 2 are listed those bands that are essentially the same for these three esters, together with bands (in about the same positions) shown by tetra-*O*-acetyl-*α*-d-lyxopyranose (compound 3). Bands shown by all four of these acetates are tentatively assumed to be independent of total configuration, whereas those shown by compounds 1 and 2 but not by both compounds 3 and 4, or by compounds 3 or 4 but not by compounds 1 and 2, may possibly be affected by configuration.

The bands shown by only one anomer of tetra-*O*-acetyl-d-xylopyranose are given in table 3, together with corresponding bands of tetra-*O*-acetyl-*α*-l-arabinopyranose. The resemblance between compounds 2 and 4 may be noted. If these “anomer-differentiating” bands have any relationship to the axial or equatorial disposition of the anomeric acetoxyl group, it may be concluded that compounds 2 and 4 have the same anomeric disposition, whereas that for compound 1 is different. If the assignment previously made [6] for the methyl glycopyranoside corresponding to *any one of these three* esters is extended to the related pyranose acetate, the conformations of the other two are deducible from the results in table 3. Thus, if the anomeric acetoxyl group of compound 2 is equatorial and that of its *α* anomer is axial, the results indicate that the anomeric acetoxyl group of tetra-*O*-acetyl-*α*-l-arabinopyranose is equatorial.

On the other hand, the spectrum of compound 3, although showing two bands in common with that of compound 2 (see table 3), bears a much greater resemblance to that of compound 1, suggesting that the crystalline material (3) may have the CA conformation. If this conclusion is correct, it indicates that the stable conformation of a fully acetylated pyranose need not necessarily be the same as the stable conformation of the corresponding fully acetylated methyl glycopyranoside. Presumably, change from the methoxyl group to the acetoxyl group at carbon atom 1 may result in a sufficiently great alteration in nonbonded attractions and repulsions to cause a change in conformation.

### 5.2. Analysis of the Spectra of Groups of Configurationally Related Pyranose Acetates, Excluding the Pentopyranose Acetates

The spectra of penta-*O*-acetyl-*α*-l-*xylo*-hexulopyranose (compound 5) and hexa-*O*-acetyl-*α*-d-*gluco*-heptulopyranose (compound 8) were compared with those of the anomers of penta-*O*-acetyl-d-glucopyranose (compounds 6 and 7). Compounds 5, 6, and 8 show the following bands not shown^2^ by compound 7: at 1312 to 1302 cm^−1^; 1174 to 1168 cm^−1^; 960 to 949 cm^−1^; 938 cm^−1^; 746 to 712 cm^−1^; 418 to 413 cm^−1^; and 395 to 394 cm^−1^. Compounds 5 and 6 show bands (not shown by compounds 7 and 8) at 1115 to 1111 cm^−1^ and 556 to 550 cm^−1^, and compound 6 does not show a band at 606 to 600 cm^−1^ that is displayed by compounds 5, 7, and 8; with these exceptions, these observations indicate that *compounds 5 and 8 resemble compound 6.* Thus, if compound 6 has an axial anomeric (acetoxyl) group, it seems likely that compounds 5 and 8 also have an axial anomeric acetoxyl group and, consequently, have an equatorial acetoxymethyl group attached to C1 of the xylopyranose ring.

The spectrum of hexa-*O*-acetyl-l-*glycero*-*β*-d-*gluco*-heptopyranose (compound 9) was now compared with those of compounds 6 and 7. The results are given in table 4, from which it may be seen that compounds 6 and 9 have 5 bands in common (column C) that are not shown by compound 7. On the other hand, compounds 7 and 9 show 2 bands (column E) not shown by compound 6, and compound 6 shows 6 bands (column D) not shown by compounds 7 and 9. These results indicate about equal similarity between compounds 7 and 9 as between compounds 6 and 9, and, hence, suggest either (a) that compound 9 adopts a non-chair conformation or a mixture of the CA and CE conformations, or (b) that, in this kind of analysis, the spectrum of a fully acetylated heptopyranose may not necessarily be intercomparable with those of the related hexopyranose acetates.

Penta-*O*-acetyl-*β*-d-mannopyranose and hexa-*O*-acetyl-d - *glycero* - *α* - l - *manno* - heptopyranose (compounds 12 and 13) show two bands (at 3003 to 2985 cm^1−^ and at 616 to 615 cm^−1^) that are not shown by penta-*O*-acetyl-*α*-d-mannopyranose (compound 11). On the other hand, compounds 11 and 13 show seven bands that are not shown by compound 12: at 1295 to 1294 cm^1−^, 1081 to 1076 cm^−1^, 839 to 837 cm^−1^, 787 to 777 cm^−1^, 475 to 466 cm^−1^, 439 to 433 cm^−1^, and 414 to 413 cm^−1^; and compound 12 shows 12 bands that are not shown by compounds 11 and 13: at 2882, 1383, 1093, 758 (this band was mentioned by Barker and coworkers [5]), 589, 520, 488, 446, 407, 377, 364, and 345 cm^−1^. These results indicate a considerable resemblance between compounds 11 and 13. Thus, if the anomeric acetoxyl group of compound 11 is axial, it seems likely that the anomeric acetoxyl group of hexa-*O*-acetyl-d-*glycero*-*α*-l-*manno*-heptopyranose (13) is also axial.

In parts II and III of this series [6,7], evidence was obtained that indicated that the methyl glycopyranosides and their acetates corresponding to the *α* and *β* anomers of hexa-*O*-acetyl-d-*glycero*-d-*gulo*-heptopyranose (15 and 16; see table 1) have the CA conformation, whereas those corresponding to penta-*O*-acetyl-*α*-d-gulopyranose (compound 14) consist of a mixture of the CA and CE conformations or adopt a non-chair conformation. In table 5,^3^ the anomer-differentiating bands of compounds 15 and 16 are compared with bands shown by compound 14. It may be seen that compound 14 shows about equal similarity to either compound 15 or 16; this conclusion suggests that crystalline penta-*O*-acetyl-*α*-d-gulopyranose (compound 14) exists as a mixture of the CA and CE conformations or as a non-chair conformation.

Finally, an assignment of conformation was sought for hexa-*O*-acetyl-d-*glycero*-*β*-D-*galacto*-heptopyranose (compound 19). In table 6, the bands differentiating between penta-*O*-acetyl-*α*-d-galactopyranose (compound 17) and its *β* anomer (compound 18) are compared with bands for compound 19. It may be seen that compound 19 shows about equal similarity to either compound 17 or 18, suggesting that crystalline hexa-*O*-acetyl-d-*glycero*-*β*-d-*gatacto*-heptopyranose (compound 19) exists as a mixture of the CA and CE conformations or as a non-chair conformation. This conclusion is surprising, as there has hitherto been no indication of such conformations for compounds having a *galacto* configuration. Possibly, as previously mentioned, the spectrum of a fully acetylated heptopyranose may not, in this kind of analysis, necessarily be intercomparable with those of the related hexopyranose acetates.

No assignment could be made for hexa-*O*-acetyl-d-*glycero*-*β*-d-*ido*-heptopyranose (compound 10), because of the lack of its *α* anomer. Similarly, no assignment could be made for penta-*O*-acetyl-*α*-d-talopyranose (compound 20), because of a lack of other pyranose acetates having the *talo* configuration.

From a study of the proton magnetic-resonance spectra of compounds 1 to 4, 6, 7, 11, 12, 14, 17, and 18, Lemieux and coworkers [11] made exactly the same assignments of anomeric disposition as we now find for these compounds (see table 1).

Many of the bands observed for the pyranose acetates cannot yet be assigned to particular vibrational modes; in sections 5.1 and 5.2, we have not been concerned with (a) which bands, arising from vibrations localized in a functional group, are relatively independent of the remainder of the molecule, or (b) which bands involve other parts of the molecule. Bands possibly attributable to specific functional groups are considered in section 5.3.

### 5.3. Other Absorption Bands

All of the compounds in this study are acetates, and their spectra all show at least one band at 1757 to 1742 cm^−1^ (C = O stretching frequency); at 1269 to 1241 cm^−1^; at 1236 to 1212 cm^−1^; at 646 to 626 cm^−1^; and at 612 to 598 cm^−1^. All of the spectra show at least one band at 2985 to 2950 cm^−1^ (or 2976 to 2941 cm^−1^; C—H stretching); and at 1449 to 1420 cm^−1^ and 1333 to 1312 cm^−1^ (C—H bending). All of the spectra show an absorption band at 1381 to 1366 cm^−1^, presumably caused by deformation of the CH_3_ groups. All of the spectra also show a band at 1148 to 1114 cm^−1^; 1105 to 1073 cm^−1^; 1073 to 1042 cm^−1^; 1036 to 1011 cm^−1^; 911 to 899 cm^−1^; 415 to 399 cm^−1^; and 377 to 366 cm^−1^ (or 370 to 353 cm^−1^). All of the spectra seem to show a weak band at 2132 to 2075 cm^−1^, probably a combination or overtone.

**Figure 1:**
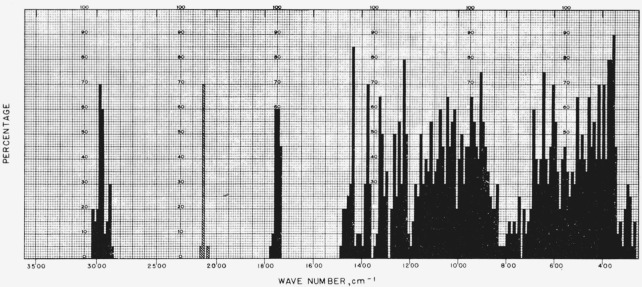
Percentage (of the 20 pyranose acetates) which showed infrared absorption at the various regions of the infrared spectrum (5000 to 250 cm^−1^).

Figure 1 gives the percentage of the 20 acetates in this study that show absorption bands in the various regions of the infrared spectrum. For the range of 5000 to 2000 cm^−1^, decrements of 20 cm^−1^ were used; and, for the range of 2000 to 250 cm^−1^, decrements of 10 cm^−1^.

## 6. Experimental Procedure

### 6.1. Preparation and Purification of the Compounds

The compounds listed in table 1 were prepared by the methods given in the references cited. Most of the compounds were prepared in the course of earlier studies on the pyranose acetates. Each acetate was recrystallized from an appropriate solvent until further recrystallization caused no change in its melting point or optical rotation.

### 6.2. Preparation of the Pellets

Samples used for photometric study consisted of pellets comprising the crystalline acetate suspended in an alkali-metal halide, exactly as previously described [10]. For the range of 5000 to 667 cm^−1^, a concentration of 0.4 mg of acetate per 100 mg of potassium chloride was used; the spectrum of compound 2 was also recorded for a film prepared on the sodium chloride window by evaporation of a carbon tetrachloride solution of the compound with a heat lamp. For the range of 667 to 250 cm^−1^, a concentration of 2 mg of acetate per 100 mg of potassium iodide was used, except for compounds 8 and 9 (3 mg per 100 mg).

### 6.3. Measurement of Infrared Absorption

**Figure 2:**
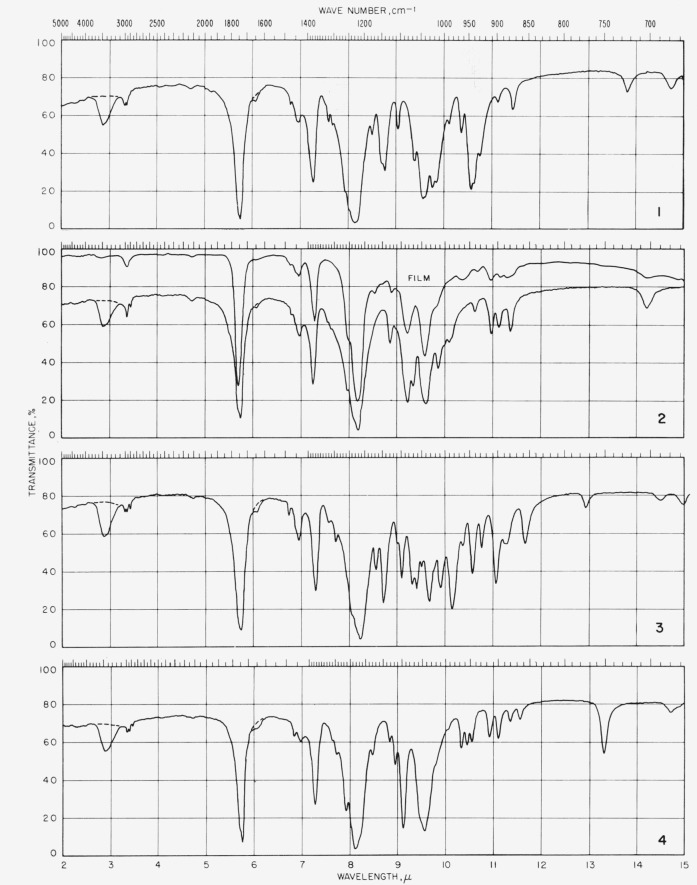

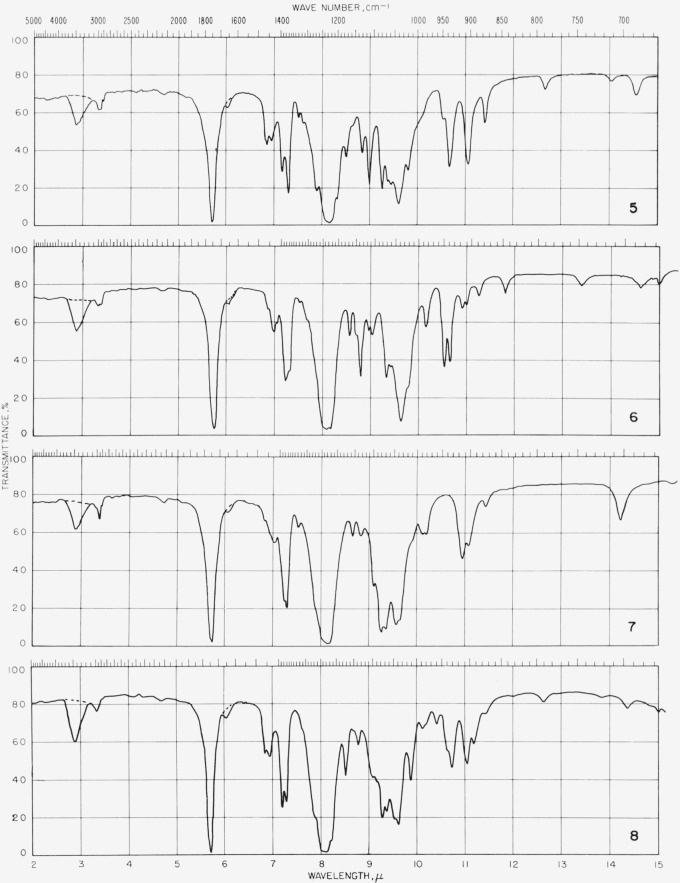

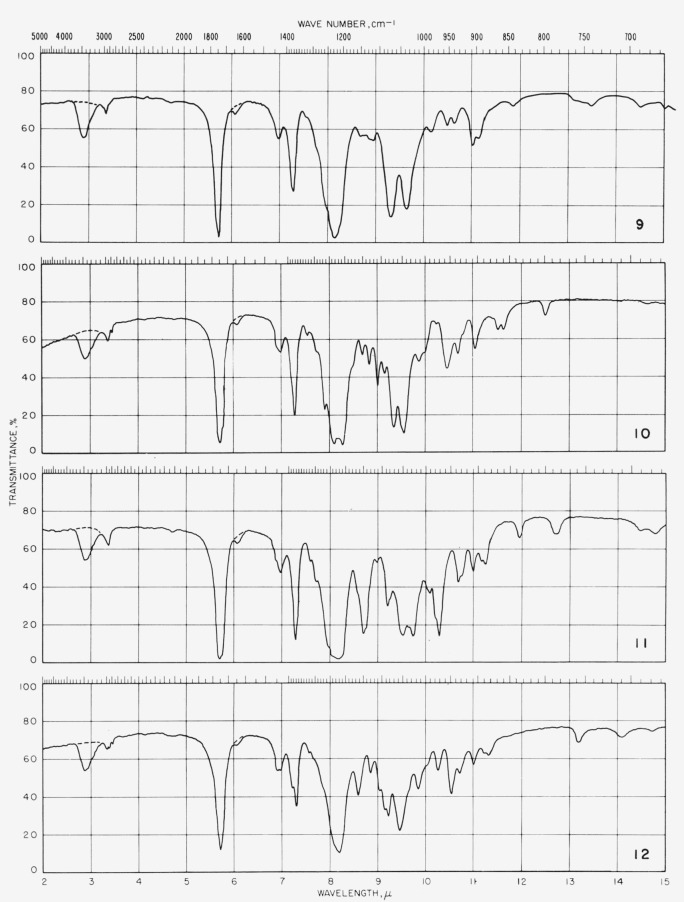

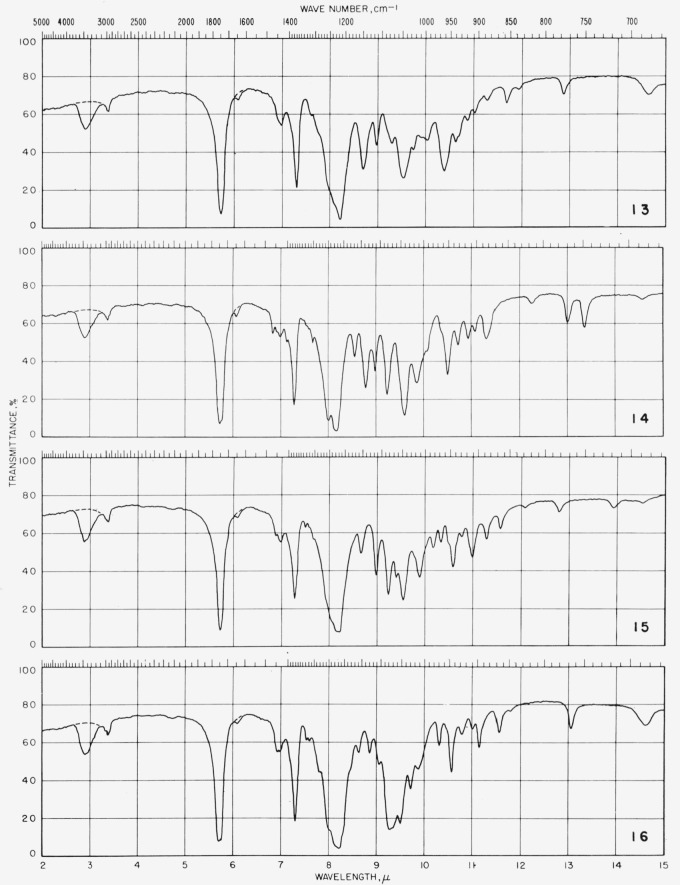

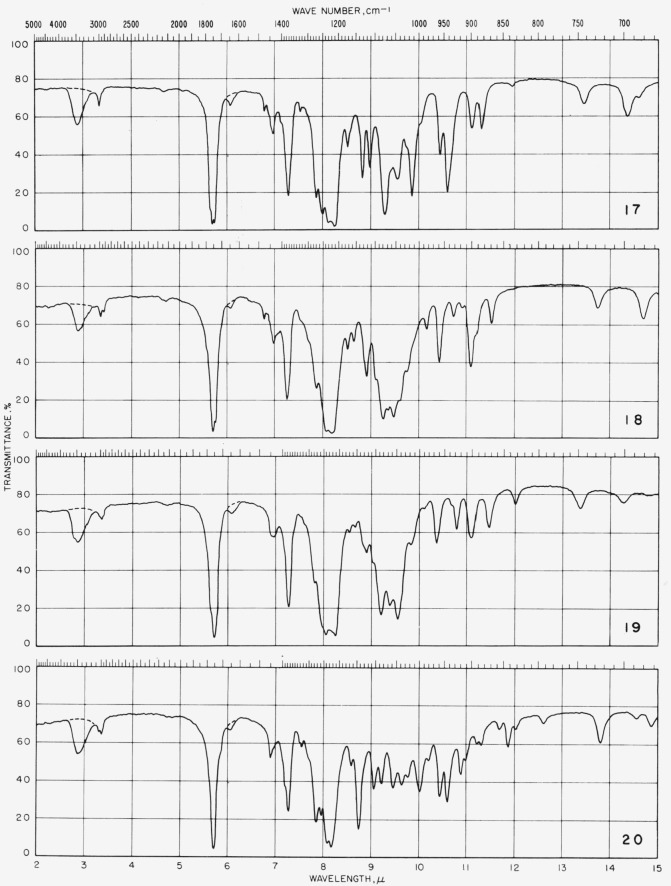

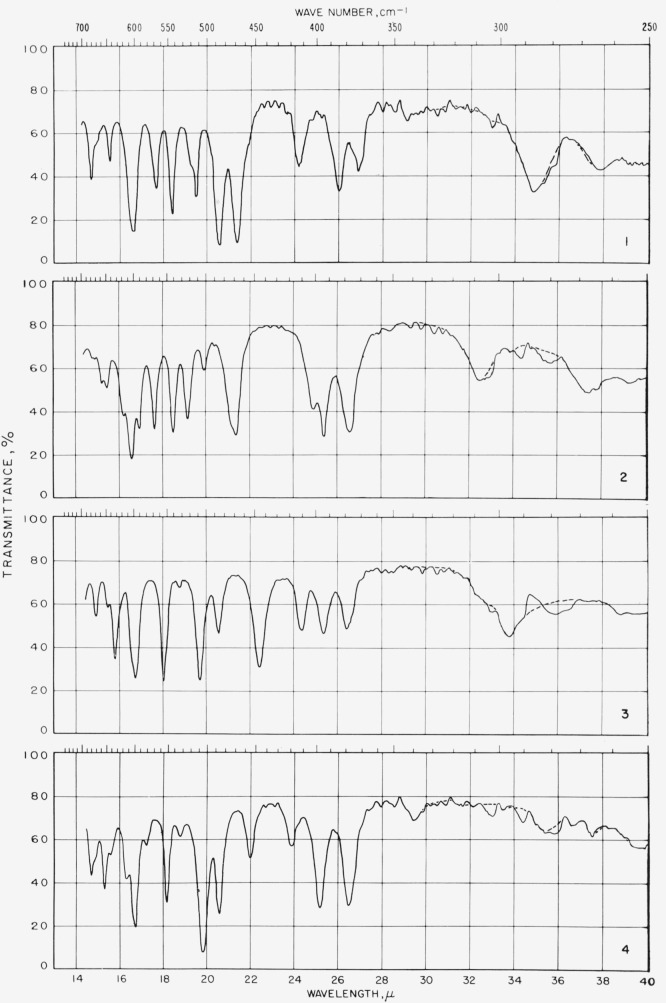
Spectrograms of materials in potassium chloride pellets. **1**, Tetra-*O*-acetyl-*α*-d-xylopyranose; **2**, tetra-*O*-acetyl-*β*-d-xylopyranose; **3**, tetra-*O*-acetyl-*α*-d-lyxopyranose; **4**, tetra-*O*-acetyl-*α*-l-arabinopyranose. **5**, penta-*O*-acetyl-*α*-l-*xylo*-hexulopyranose; **6**, penta-*O*-acetyl-*α*-d-glucopyranose; **7**, penta-*O*-acetyl-*β*-d-glucopyranose; **8**, hexa-*O*-acetyl-*α*-d-*gluco*-heptulopyranose. **9**, hexa-*O*-acetyl-l-*glycero*-*β*-d-*gluco*-heptopyranose; **10**, hexa-*O*-acetyl-d-*glycero*-*β*-d-*ido*-heptopyranose; **11**, penta-*O*-acetyl-*α*-d-mannopyranose; **12**, penta-*O*-acetyl-*β*-d-mannopyranose. **13**, hexa-*O*-acetyl-d-*glycero*-*α*-l-*manno*-heptopyranose; **14**, penta-*O*-acetyl-*α*-d-gulopyranose; **15**, hexa-*O*-acetyl-d-*glycero*-*α*-d-*gulo*-heptopyranose; **16**, hexa-*O*-acetyl-d-*glycero*-*β*-d-*gulo*-heptopyranose. **17**, penta-*O*-acetyl-*α*-d-galactopyranose; **18**, penta-*O*-acetyl-*β*-d-galactopyranose; **19**, hexa-*O*-acetyl-d-*glycero*-***β***-d-*galacto*-heptopyranose; **20**, penta-*O*-acetyl-*α*-d-talopyranose.

**Figure 3:**
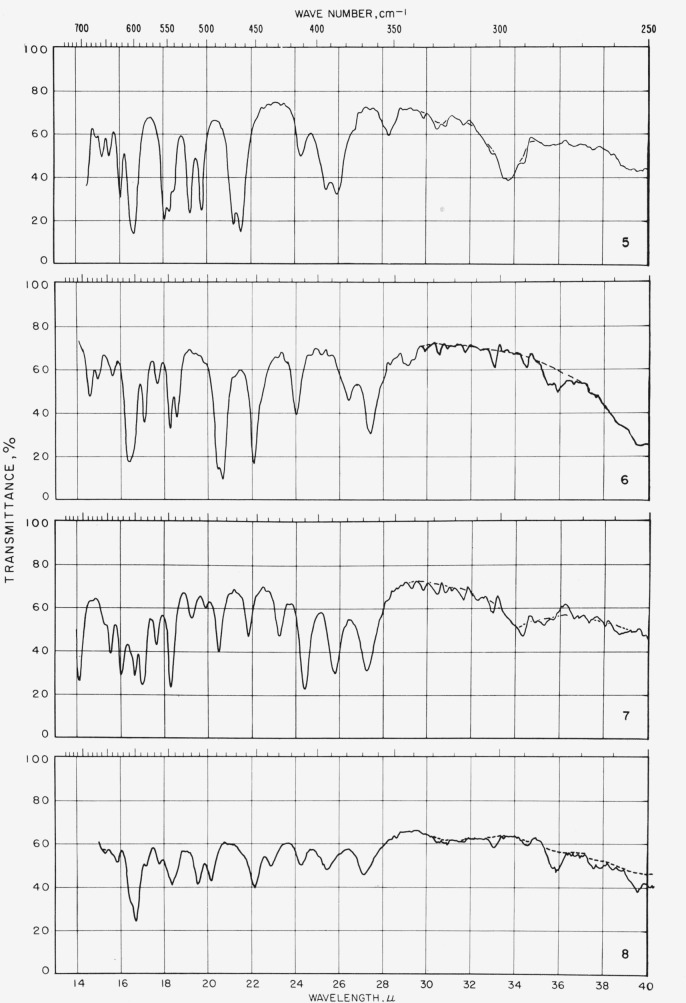

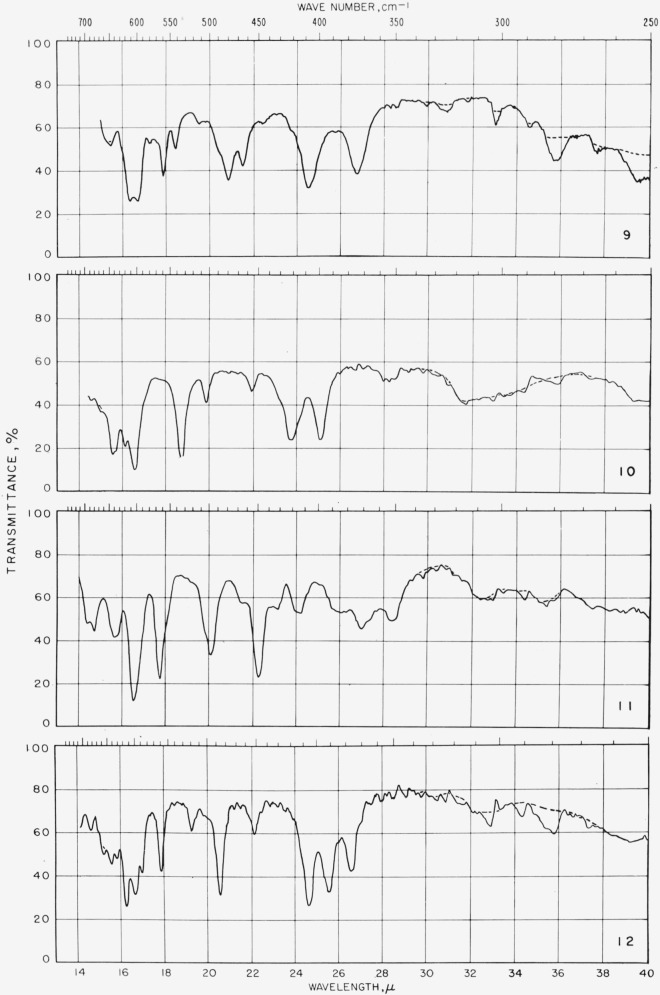

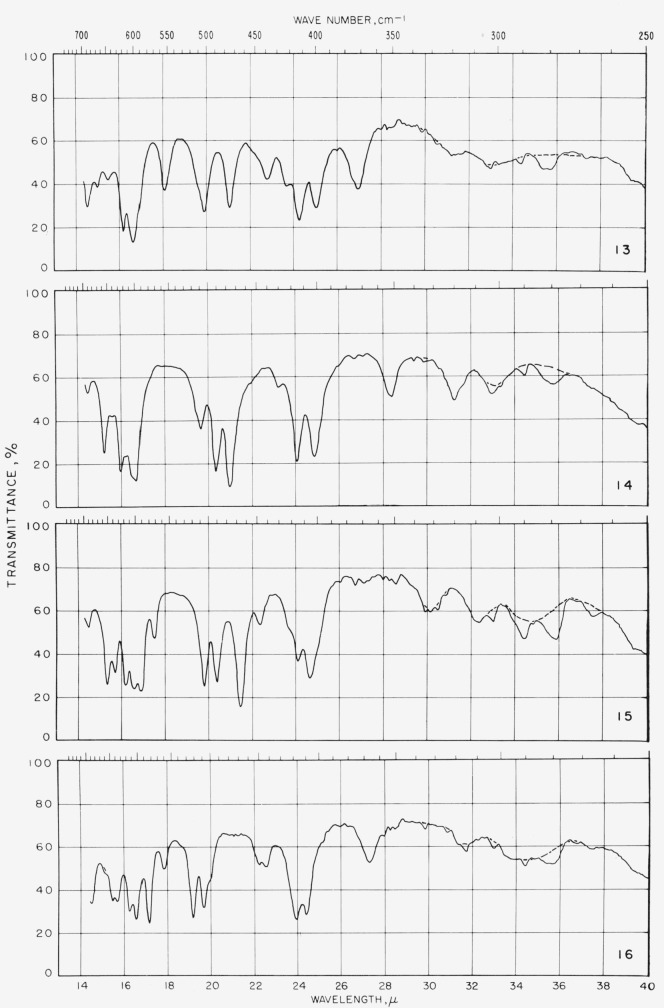

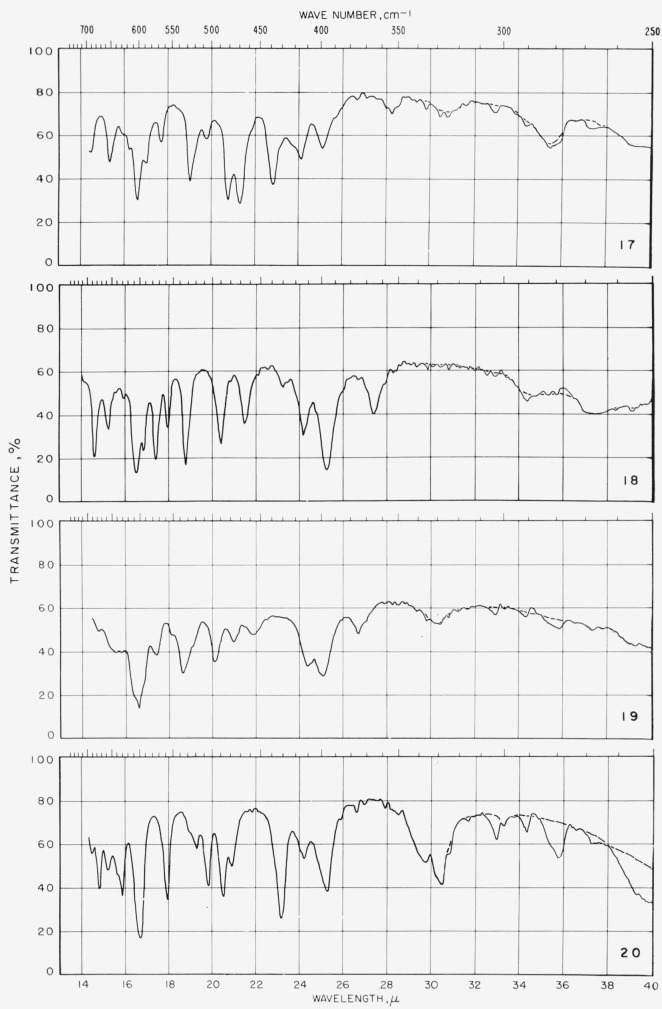
Spectrograms of materials in potassium iodide pellets. **1**, Tetra-*O*-acetyl-*α*-d-xylopyranose; **2**, tetra-*O*-acetyl-*β*-d-xylopyranose; **3**, tetra-*O*-acetyl-*α*-d-lyxopyranose; **4**, tetra-*O*-acetyl-*α*-l-arabinopyranose. **5**, penta-*O*-acetyl-*α*-l-*gylo*-hexulopyranose; **6**, penta-*O*-acetyl-*α*-d-glucopyranose; **7**, penta-*O*-acetyl-*β*-d-glucopyranose; **8**, hexa-*O*-acetyl-*α*-d-*gluco*-heptulopyranose. **9**, hexa-*O*-acetyl-l-*glycero*-*β*-d-*gluco*-heptopyranose; **10**, hexa-O-acetyl-d-*glycero*-*β*-d-*ido*-heptopyranose; **11**, penta-*O*-acetyl-*α*-d-mannopyranose; **12**, penta-*O*-acetyl-*β*-d-mannopyranose. **13**, hexa-*O*-acetyl-d-*glycero*-*α*-l-*manno*-heptopyranose; **14**, penta-*O*-acetyl-*α*-d-gulopyranose; **15**, hexa-*O*-acetyl-d-*glycero*-*α*-d-*gulo*-heptopyranose; **16**, hexa-*O*-acetyl-d-*glycero*-*β*-d-*gulo*-heptopyranose. **17**, penta-*O*-acetyl-*α*-d-galactopyranose; **18**, penta-*O*-acetyl-*β*-d-galactopyranose; **19**, hexa-*O*-acetyl-d-*glycero*-*β*-d-*galacto*-heptopyranose; **20**, penta-*O*-acetyl-*α*-d-talopyranose

The spectrograms^4^ are shown in figures 2 and 3; they were recorded with a Perkin-Elmer Model 21 (double-beam) spectrophotometer equipped with a prism of sodium chloride (for the range of 5000 to 667 cm^−1^) and of cesium bromide (for the range of 667 to 250 cm^−1^), as previously described [10].

Some absorption attributable to water (in the compound, the alkali halide, or both) was observed at 3448 and 1639 cm^−1^ and, attributable to atmospheric water vapor, in the far-infrared curves. These regions are drawn on the spectrograms with dashed lines which are not to be interpreted quantitatively.

### 6.4. Spectra Measured Under Different Conditions

The spectra of 17 of the pyranose acetates (compounds 1 to 7 and 10 to 19) had previously been measured [3] in carbon tetrachloride or chloroform and in either carbon disulfide or dioxane. Since the infrared absorption spectra of crystalline materials show more bands than the spectra of the same compounds in solution, a larger number of bands were available for correlations than in the previous study [3].

In addition, the spectra of 13 acetylated aldopyranosides had been recorded for the solid phase [7] and for solutions [3]. The spectra for the solid and the liquid phases of these 30 compounds are, in all cases, *in striking agreement* (after making allowance for the sharpening of the bands for the crystalline samples). This observation suggests that, if the geometry of the molecule is reflected in its vibration spectrum, each of these compounds adopts the same conformation(s) in the solid phase as it does in solution.

## Figures and Tables

**Table 1:** Compounds measured, stable conformations, and index to spectrograms

Code^a^	Compound	Reference	Stable conformation^b^				Spectrogram
			Assignment for methyl glycopyranoside^c^	Assignment for acetylated methyl glycoside d	Present assignment^e^	Anomeric disposition^e^	
							
12.11121	Tetra-*O*-acetyl-*α*-d-xylopyranose	1	CA	CA	CA	*a*	1
12.11221	Tetra-*O*-acetyl-*β*-d-xylopyranose	1	CA	CA	CA	*e*	2
12.12521	Tetra-*O*-acetyl-*α*-d-lyxopyranose	2	CA+CE; nonchair.	CA+CE; nonchair.	CA	*a*	3
12.13421	Tetra-*O*-acetyl-*α*-l-arabinopyranose	3	CE	…………………………………………………………………	CE	*e*	4
12.71121	Penta-*O*-acetyl-*α*-*xylo*-hexulopyranose	4	…………………………………………	…………………………………………………………………	CA	*a*	5
12.21121	Penta-*O*-acetyl-*α*-d-glucopyranose	5	CA	CA	CA	*a*	6
12.21221	Penta-*O*-acetyl-*β*-d-glucopyranose	6	CA	CA	CA	*e*	7
12.81121	Hexa-*O*-acetyl-*α*-d-*gluco*-heptulopyranose	7	…………………………………………	…………………………………………………………………	CA	*a*	8
12.41221	Hexa-*O*-acetyl-l-*glycero*-*β*-d-*gluco*-heptopyranose	8	…………………………………………	…………………………………………………………………	CA+CE; nonchair.	*a*+*e; a, e*, or *q*	9
12.35?21	Hexa-*O*-acety d-*glycero*-*β*-d-*ido*-heptopyranose	9	…………………………………………	…………………………………………………………………	…………………	…………………	10
12.22121	Penta-*O*-acetyl-*α*-d-mannopyranose	10	CA	CA	CA	*a*	11
12.22221	Penta-*O*-acetyl-*β*-d-mannopyranose	11	…………………………………………	CA	CA	*e*	12
12.42121	Hexa-*O*-acetyl-d-*glycero*-*α*-l-*manno*-heptopyranose.	12	CA	…………………………………………………………………	CA	*a*	13
12.26521	Penta-*O*-acetyl-*α*-d-gulopyranose	9	CA+CE; nonchair.	CA+CE; nonchair.	CA+CE; nonchair.	*a+e; a, e*, or *q*	14
12.36121	Hexa-*O*-acetyl-d-*gycero*-*α*-d-*gulo*-heptopyranose	13	CA	CA	CA	*a*	15
12.36221	Hexa-*O*-acetyl-d-*glycero*-*β*-d-*gul*o-heptopyranose	13	CA	CA	CA	*e*	16
12.23121	Penta-*O*-acetyl-*α*-d-galactopyranose	14	CA	CA	CA	*a*	17
12.23221	Penta-*O*-acetyl-*β*-d-galactopyranose	14	CA	CA	CA	*e*	18
12.33521	Hexa-*O*-acetyl-d-*glycero*-*β*-d-*galacto*-heptopyranose.	15	…………………………………………	…………………………………………………………………	CA+CE; nonchair.	*a*+*e; a, e*, or *q*	19
12.24?21	Penta-*O*-acetyl-*α*-d-talopyranose	16	…………………………………………	…………………………………………………………………	…………………	…………………	20

a The third figure after the point was inserted after the present conclusions as to conformation had been reached.

b Named by the system of H. S. Isbell and R. S. Tipson, Science **130**, 793 (1959); J. Research NBS **64A**, 171 (1960).

c Assignment made by R. S. Tipson and H. S. Isbell, J. Research NBS **64A**, 239 (1960).

d Assignment made by R. S. Tipson and H. S. Isbell, J. Research NBS **64A**, 405 (1960).

e After accepting several of the assignments for the corresponding unacetylated or acetylated methyl glycopyranosides (see text).

*References for* Table 1

1 C. S. Hudson and J. M. Johnson, J. Am. Chem. Soc. **37**, 2748 (1915).

2 P. A. Levene and M. L. Wolfrom, J. Biol. Chem. **78**, 525 (1928).

3 C. S. Hudson and J. K. Dale, J. Am. Chem. Soc. **40**, 992 (1918).

4 H. H. Schlubach and G. Graefe, Liebigs Ann. Chem. **532**, 211 (1937).

5 A. Georg, Helv. Chim. Acta **12**, 261 (1929).

6 C. S. Hudson and J. K. Dale, J. Am. Chem. Soc. **37**, 1264 (1915).

7 W. C. Austin, J. Am. Chem. Soc. **54**, 1925 (1932).

8 H. L. Frush and H. S. Isbell, unpublished results. The optical rotation of the compound was equal in magnitude, but opposite in sign, to that of its enantiomorph [R. Hann and C. S. Hudson, J. Am. Chem. Soc. **59**, 548 (1937)], and the two compounds had the same melting point.

9 H. L. Frush and H. S. Isbell, J. Research NBS **35**, 111 (1945) RP1663.

10 C. S. Hudson and J. K. Dale, J. Am. Chem. Soc. **37**, 1280 (1915).

11 D. H. Brauns, J. Research NBS **7**, 573 (1931) RP358.

12 H. S. Isbell, F. A. Smith, E. C. Creitz, H. L. Frush, J. D. Moyer, and J. E Stewart, J. Research NBS **59**, 41 (1957) RP2772.

13 C. S. Hudson and E. Yanovsky, J. Am. Chem. Soc. **38**, 1575 (1916).

14 C. S. Hudson and H. O. Parker, J. Am Chem. Soc. **37**, 1589 (1915).

15 E. Montgomery and C. S. Hudson, J. Am. Chem. Soc. **56**, 2463 (1934).

16 W. W. Pigman and H. S. Isbell, J. Research NBS **19**, 189 (1937) RP1021.

**Table 2:** Bands (cm^−1^) shown by both anomers of tetra-*O*-acetyl-d-xylopyranose (compounds 1 and 2) and by tetra-*O*-acetyl-*α*-l-arabinopyranose (4), and positionally corresponding bands of tetra-*O*-acetyl-*α*-d-lyxopyranose (3)

Tetra-*O*-acetyl-d-xylopyranoses		Tetra-*O*-acetyl-*α*-l-arabino-pyranose	Tetra-*O*-acetyl-*α*-d-lyxopyranose
1	2	4	3
Possibly non-configurational bands			
2959	2967	2976	2967
2915	2890	2924	2899
2114	2114	2114	2105
1739	1742	1736	1742
1473	1473	1462	1484
1439	1437	1433	1439
1377	1381	1374	1377
1230	1235	1233	1227
1178	1172	1181	1170
1105	1085	1096	1099
1046	1041	1045	1052
1026	1028(?)	a1024	1021
989	991	995	985
965	962	967	963
947, a942	b941	b948	946
899	b899	b900	903, 889
875	b880	b880	885
679	703	679	690
673	671	671	670
643	646	643	647
601	602	600	599
567	568	553	556
545	542	536	534
515	502	506	508
488	491	489	488
398	394	399	395
372	377	379	379
365	364	366	369, 364
358	359	359	358
351	353	351	354
344	342	341	342
288	309	283	297

Bands possibly affected by configuration and conformation			
………………………………	…………………………	2874	………………………………………………………………
1751	1757	1751	………………………………………………………………
1330	1326	1332	………………………………………………………………
1304	1312	1309	………………………………………………………………
………………………………	…………………………	1295	1295
1261	1258	1263	………………………………………………………………
1142	1130	1133	………………………………………………………………
………………………………	…………………………	1116	111
1066	1071	…………………………………………	1073, 1064
a1016	1014	…………………………………………	a1009
………………………………	…………………………	956	………………………………………………………………
930	935	…………………………………………	929
………………………………	…………………………	b865	857
………………………………	…………………………	b751	774
520	523	…………………………………………	………………………………………………………………
458	459	457	………………………………………………………………
448 (?)	451(?)	…………………………………………	447
443 (?)	444 (?)	442(?)	………………………………………………………………
437(?)	436(?)	435(?)	………………………………………………………………
430 (?)	429 (?)	…………………………………………	………………………………………………………………
424 (?)	423 (?)	421	………………………………………………………………
404	402	…………………………………………	………………………………………………………………
264	268	274, 267	………………………………………………………………

a These bands were mentioned by R. L. Whistler and L. R. House [4].

b These bands were mentioned by S. A. Barker and coworkers [5]—for the enantiomorphs of the compounds in tables 2 and 3.

**Table 3:** Bands (cm^−1^) shown by only one anomer of the tetra-*O*-acetyl-d-xylopyranoses (compounds 1 and 2) and by tetra-*O*-acetyl-*α*-l-arabinopyranose (4), compared with bands for tetra-*O*-acetyl-*α*-d-lyxopyranose (3)

1	2	4	3
			
2994	……………	……………	3003
1319	……………	……………	1321
1247	……………	……………	1242
1149	……………	……………	1148
1038	……………	……………	1033
724	……………	……………	……………
469	……………	……………	……………
414	……………	……………	412
386	……………	……………	……………
			
……………	1222	1225, 1218	1217
……………	a912	a915	……………
……………	656	655	……………
……………	616	615	633
……………	591	582	……………

a See footnote b to table 2.

**Table 4:** Comparison^a^ of absorption bands (cm^−1^) shown by the anomers of penta-*O*-acetyl-d-glycopyranose (compounds 6 and 7) and by hexa-*O*-acetyl-l-*glycero*-*β*-d-*gluco*-heptopyranose (9)

A		B	C		D	E	
6	7	9	6	9	6	7	9
							
1456	1464	1215(?)	1115	1119	1414	600	597
1383	1381	761	b950	955	1302	442	445
1105	1098	…………	b,c938	941	1238	…………	…………
1062	1068		b[846	d844]	1168		
1038	1044		b746	742	550		
c1022	1010		418	422	399, 395		
b917	b913		…………	…………	…………		
666	654 (?)						
474	475						
434, 427	431						
366	368						

a Key: A. Bands shown by compounds 6 and 7, but not by 9. B. Bands shown by compound 9, but not by 6 and 7. C. Bands shown by compounds 6 and 9, but not by 7. D. Bands shown by compound 6, but not by 7 and 9. E. Bands shown by compounds 7 and 9, but not by 6.

b See footnote b to table 3.

c See footnote a to table 2.

d Compound 7, in carbon tetrachloride, shows a band at 855 cm^−1^ [3].

**Table 5:** Bands (cm^−1^) shown^a^ by only one anomer of hexa-*O*-acetyl-d-glycero-d-gulo-heptopyranose (compounds 15 and 16), compared with bands for penta-*O*-acetyl-*α*-d-gulo-pyranose (14)

A		B	C		D
14	15	16	14	16	15
					
1462	1453	3003	2941	2941	1366
1304	1304	1280	1443	1443	1064
1114	1114	1205	1318	1319	781
994	983	1105	b1170	1186	718
952	952	1030	1139	1130	
884	887	848	903	899	
816	829	523	769	766	
477	462	444			

a Key: A. Bands shown by compounds 14 and 15, but not by 16. B. Bands shown by compound 16, but not by 14 and 15. C. Bands shown by compounds 14 and 16, but not by 15. D. Bands shown by compound 15, but not by 14 and 16.

b See footnote a to table 2.

**Table 6:** Comparison^a^ of absorption bands (cm^−1^) shown by the penta-*O*-acetyl-d-galactopyranoses (compounds 17 and 18) and by hexa-*O*-acetyl-d-glycero-*β*-d-galacto-heptopyranose (19)

A		B	
17	19	18	19
			
1779	1770	1156	1155
1250	1252	b1122	1124
1133	b1131	1068	1065
837	832	c916	927
696	700	c869	873
615	610	641	641
		575	573
		366	370

a Key: A. Bands shown by compounds 17 and 19, but not by 18. B. Bands shown by compounds 18 and 19, but not by 17.

b See footnote a to table 2.

c See footnote b to table 2.

## Footnotes

1 Figures in brackets indicate the literature references at the end of this paper. The references for table 1 are given at the end of the table.

2 Barker and coworkers [5] originally reported bands at 949 and 938 cm^−1^ for compound 7. In a private communication from S. A. Barker and R. Stephens (August 12, 1960), we learn that they have since recorded the spectrum of a purified sample of compound 7 and find no absorption between 978 and 911 cm^−1^.

3 To conserve space, bands which do not differentiate between anomers have been omitted from tables 5 and 6.

4 A few bands that show clearly in the original spectrograms are difficult to see in the published spectra, because of the reduction in size.
